# Binding of Protein Kinase Inhibitors to Synapsin I Inferred from Pair-Wise Binding Site Similarity Measurements

**DOI:** 10.1371/journal.pone.0012214

**Published:** 2010-08-16

**Authors:** Enrico De Franchi, Claire Schalon, Mirko Messa, Franco Onofri, Fabio Benfenati, Didier Rognan

**Affiliations:** 1 Department of Neuroscience and Brain Technologies, The Italian Institute of Technology, Genova, Italy; 2 Structural Chemogenomics, Laboratory of Therapeutic Innovation, CNRS UMR 7200, Université de Strasbourg, Illkirch, France; 3 Department of Experimental Medicine, University of Genova and Istituto Nazionale di Neuroscienze, Genova, Italy; The Scripps Research Institute, United States of America

## Abstract

Predicting off-targets by computational methods is getting increasing importance in early drug discovery stages. We herewith present a computational method based on binding site three-dimensional comparisons, which prompted us to investigate the cross-reaction of protein kinase inhibitors with synapsin I, an ATP-binding protein regulating neurotransmitter release in the synapse. Systematic pair-wise comparison of the staurosporine-binding site of the proto-oncogene Pim-1 kinase with 6,412 druggable protein-ligand binding sites suggested that the ATP-binding site of synapsin I may recognize the pan-kinase inhibitor staurosporine. Biochemical validation of this hypothesis was realized by competition experiments of staurosporine with ATP-γ^35^S for binding to synapsin I. Staurosporine, as well as three other inhibitors of protein kinases (cdk2, Pim-1 and casein kinase type 2), effectively bound to synapsin I with nanomolar affinities and promoted synapsin-induced F-actin bundling. The selective Pim-1 kinase inhibitor quercetagetin was shown to be the most potent synapsin I binder (IC50  = 0.15 µM), in agreement with the predicted binding site similarities between synapsin I and various protein kinases. Other protein kinase inhibitors (protein kinase A and chk1 inhibitor), kinase inhibitors (diacylglycerolkinase inhibitor) and various other ATP-competitors (DNA topoisomerase II and HSP-90α inhibitors) did not bind to synapsin I, as predicted from a lower similarity of their respective ATP-binding sites to that of synapsin I. The present data suggest that the observed downregulation of neurotransmitter release by some but not all protein kinase inhibitors may also be contributed by a direct binding to synapsin I and phosphorylation-independent perturbation of synapsin I function. More generally, the data also demonstrate that cross-reactivity with various targets may be detected by systematic pair-wise similarity measurement of ligand-annotated binding sites.

## Introduction

For long, drug designers had been focusing on a single macromolecular target and a single or very few chemical series [1]. The selectivity of preclinical candidates for the intended target was only addressed relatively at a late stage by profiling the compound against neighboring targets (e.g. receptor subtypes). Therefore, a significant attrition rate in clinical trials in the last decades [2] was due to the unexpected binding of drug candidates to additional targets (off-targets [3] or anti-targets [4]) resulting in dubious pharmacological activities, side effects and sometimes adverse drug reactions [5]. Remarkable advances in structural genomics [6], [7] and diversity-oriented chemistry [8], [9] have changed these practices. On the biological side, the Protein Data Bank [10] which stores publicly available three-dimensional (3-D) structures of macromolecules currently stores over 65 000 entries. Outstanding efforts of structural genomic consortia to complete the structural proteome let us anticipate an acceptable coverage of the UniProt database [11] in only 15 years [7]. On the chemical side, about 27 million unique structures and 435 000 bioactivity screens are available in the PubChem repository [12]. Mapping pharmacological space in 2006 [13] resulted in more than 1 300 targets with significant affinities (<10 µm) for small molecular-weight ligands. Global chemogenomic approaches [14] targeting arrays of ligands (rows) and proteins (columns) to generate huge two-dimensional binding matrices enlarge our vision of how chemical and biological spaces match [15]. Experimental chemogenomics is however expensive, time-consuming and addresses only a restricted subset of chemical (a few thousand ligands) and biological space (a few hundred targets). Combining bio- and chemoinformatic structural approaches [13], [16], [17] to fill chemogenomic matrices presents the noticeable advantage to considerably extend space coverage and limit the number of supporting experimental validations. Predicting missing data in chemogenomic matrices can be operated on a column-by-column (virtual screening of ligand libraries [18]) or on a row-by-row basis (virtual profiling of a ligand against an array of targets [19]). Two main computational strategies are possible to profile a ligand against a panel of putative targets. On one side, ligand-based methods [9], [20], [21] aim at comparing chemical descriptors of biologically-characterized ligands to transfer the target annotation of similar molecules to the query ligand. To overcome structure-activity cliffs [22] and gain statistical relevance, it is preferable to compare sets of diverse ligands. Diverse descriptors and methods have already been validated on existing data [23], [24], [25]. This approach led to the discovery of several off-targets for known drugs [20], [21]. However, pure ligand-based methods have two main drawbacks : (i) they are restricted by the incomplete coverage of target space by known ligands and thus cannot be applied to orphan proteins, (ii) the dogma stating that chemical similarity implies biological similarity is only true in 30% of test cases [26].

On the other side, target-based approaches can also be used to profile a ligand of interest. The most straightforward method is docking a ligand to a collection of protein cavities [27], [28], [29], [30]. This strategy led to the identification of novel targets for existing ligands [5], [31], [32], [33], [34] or for a novel chemotype [35]. Molecular docking is however notoriously hampered by the lack of reliable binding free energy scoring functions [36] and the extreme difficulty to automate the set-up of heterogeneous binding sites [30]. Acknowledging that similar binding sites should recognize similar ligands, a structure-based alternative to docking, is the 3-D comparison of protein-ligand binding sites [37]. As for ligand-based methods, structural descriptors of ligand-characterized binding sites are used to transfer the ligand annotation of putative targets to the query binding site. The method requires a proper metric to compare binding sites in 3-D space and should be able to detect global as well as local similarities among unrelated 3-D structures. Despite the numerous methods described for measuring 3-D similarities between protein-ligand binding sites [37], there are still very few reports of predictive target identifications by systematic binding site comparisons (for a recent review see [38]). We herewith present a predictive study supported by biochemical and functional studies that successfully assigns an unexpected target (synapsin I) to a series of therapeutically important bioactive ligands (serine/threonine protein kinase inhibitors).

## Results

**Figure 1:**
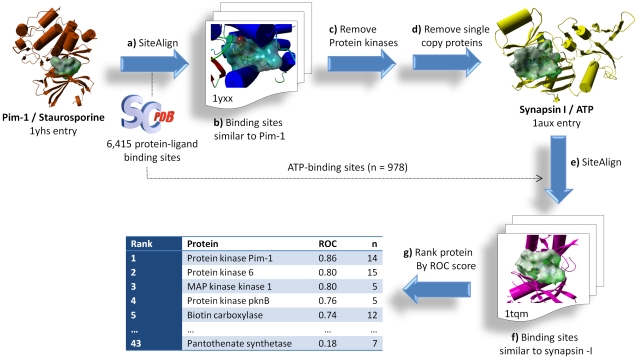
Computational protocol used to detect local similarities between ATP-binding sites in Pim-1 kinase and Synapsin I. a) The ATP-binding site in Pim-1 kinase (occupied by the ligand staurosporine) is compared with SiteAlign [41] (step a) to 6,415 binding sites stored in the sc-PDB database . Among top scoring entries (step b), synapsin I is the only protein not belonging to the protein kinase target family (step c) and present in numerous copies (step d). A systematic SiteAlign comparison (e) of the ATP-binding site in synapsin-I with 978 other ATP-binding sites suggest that some but not all ATP-binding sites of protein kinases (steps f, g) are similar to that of synapsin I.

The full computational protocol used to detect binding site similarity between synapsin-I and some protein kinases is displayed in **Fig.1**. Over 6,000 druggable protein-ligand binding sites from the sc-PDB database [39] were screened (step a, **Fig. 1**) for their similarity to the ATP-binding site of Pim-1 kinase (PDB entry 1yhs with bound inhibitor staurosporine) using the previously described SiteAlign algorithm [40]. From the list of similar binding sites (step b), ATP-binding sites of protein kinases were removed due to their obvious similarity (step c) and only proteins with at least 2 copies (two different sc-PDB entries) were kept (step d). Synapsin-I is the only hit (PDB entries 1aux and 1px2) and was used in a second similarity screen (step e), yet as a reference, for finding among ATP-binding sites which are similar. Among the list of possible hits (step f), the sc-PDB entries were ranked by decreasing similarity to 1aux and corresponding proteins were ranked (step g) according to a Receiver Operating Characteristic (ROC) classifying scheme [40] from the statistically most similar (Pim-1 kinase) to the least similar (panthothenate synthase).

Details of the multi-step protocol and the subsequent experimental validations will be described from here on.

### ATP-binding sites of synapsin I and of Pim-1 kinase share strikingly similar features

**Figure 2:**
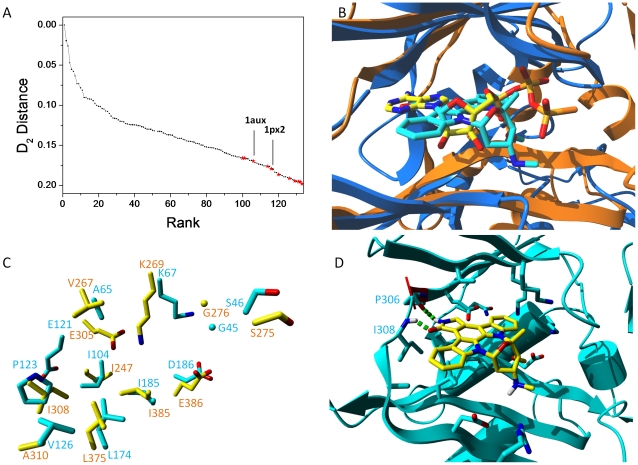
Similarity of the ATP-binding sites of Pim-1 kinase and of synapsin I. **A.** SiteAlign [41] virtual screening of 6 415 sc-PDB binding sites to the staurosporine binding site of human Pim-1 kinase (1yhs). Entries exhibiting similar binding site properties (d2 <0.20) are ranked by decreasing d2 distance to the query. ATP-binding sites of protein kinases are displayed by dark circles, other binding sites by red stars. Two ATP-binding sites of synapsin I are labeled by their Protein Data Bank entry name (1aux, 1px2). **B.** 3-D SiteAlign alignment of human Pim-1 (1yhs, blue ribbons) bound to staurosporine (cyan sticks) and of bovine synapsin I (1aux, orange ribbons) bound to adenosine 5′-diphosphate monothiophosphate (yellow sticks). Nitrogen, oxygen and sulfur atoms of bound ligands are colored in blue, red and orange, respectively. **C.** 11 matching residues between the ATP-binding sites of Pim-1 (1yhs, cyan sticks) and synapsin I (yellow sticks). Residues are labeled according to the PDB residue numbering at their Cα atom. **D.** Putative docking pose of staurosporine (yellow sticks) to the bovine synapsin I X-ray structure (1aux, cyan ribbons). Protein-ligand hydrogen bonds are displayed by green dots.

In benchmarking our 3-D binding site comparison algorithm (SiteAlign) [41], we have previously compared ATP-binding sites of protein kinases with other druggable protein-ligand cavities from the sc-PDB database [39]. The ATP-binding site of synapsin I was predicted to be similar to that of a pan-kinase inhibitor (staurosporine) [42] with the proto-oncogene Pim-1 serine/threonine protein kinase (**Fig. 2A**).

Protein kinases catalyze the reversible phosphorylation of proteins and constitute a family of macromoleuclar targets of utmost interest for their central implication in signal transduction pathways [43]. Thanks to existing X-ray structures [44], various inhibitors competing with the ATP substrate and exhibiting different selectivity profiles [42] towards the 518 human protein kinases have been designed, and some of them have reached the market as anti-cancer drugs [45].

Synapsin I belongs to an evolutionary conserved family of neuron-specific, synaptic vesicle-associated phosphoproteins involved in the regulation of neurotransmitter release, synaptic plasticity and synaptogenesis [46], [47], [48]. Synapsin isoforms are composed of a mosaic of shared and individual domains, among which the amino-terminal domain A and the large central domain C are the most conserved across isoforms and species [49], [50]. The crystal structure of the recombinant C domain [51] or ABC domains [52] of synapsin I revealed a high similarity to proteins of the ATP-grasp superfamily, notably glutathione synthase, and the presence of tightly associated dimers that can associate in a tetramer. Indeed, *in vitro* studies showed that ATP binds to all synapsins and that synapsins form homo- and hetero-oligomers [53], [54], [55]. The binding of ATP affects the oligomerization state of the synapsin ABC domains [52] and the interaction of synapsin I with the immunophilin cyclophillin B [56]. Moreover, synapsin I is a major presynaptic substrate of distinct protein kinases including PKA, CaM kinases I/II/IV, MAPK/Erk, cdk5, PAK and Src [46], [57], [58], [59], [60] that regulates synaptic vesicle trafficking, synaptic plasticity and neuronal development in a phosphorylation-dependent fashion [61], [62], [63], [64], [65], [66], [67], [68]. Based on the structural similarity between the crystal structure of the synapsin C domain with ATPases, a highly evolutionary conserved ATP binding site has been mapped in domain C [50], [51] and found to bind ATP with nanomolar affinity in a Ca^2+^-dependent manner [54]. Although very few data exist in the literature, it has been reported that a domain C peptide corresponding to a sequence between the ATP binding site and the Ca^2+^-binding site specifically inhibits the binding of synapsin I to F-actin [69]. ATP binding to synapsin I facilitates the transition from dimer to tetramer [52] and inhibits cyclophilin B binding [56].

**Figure 3:**
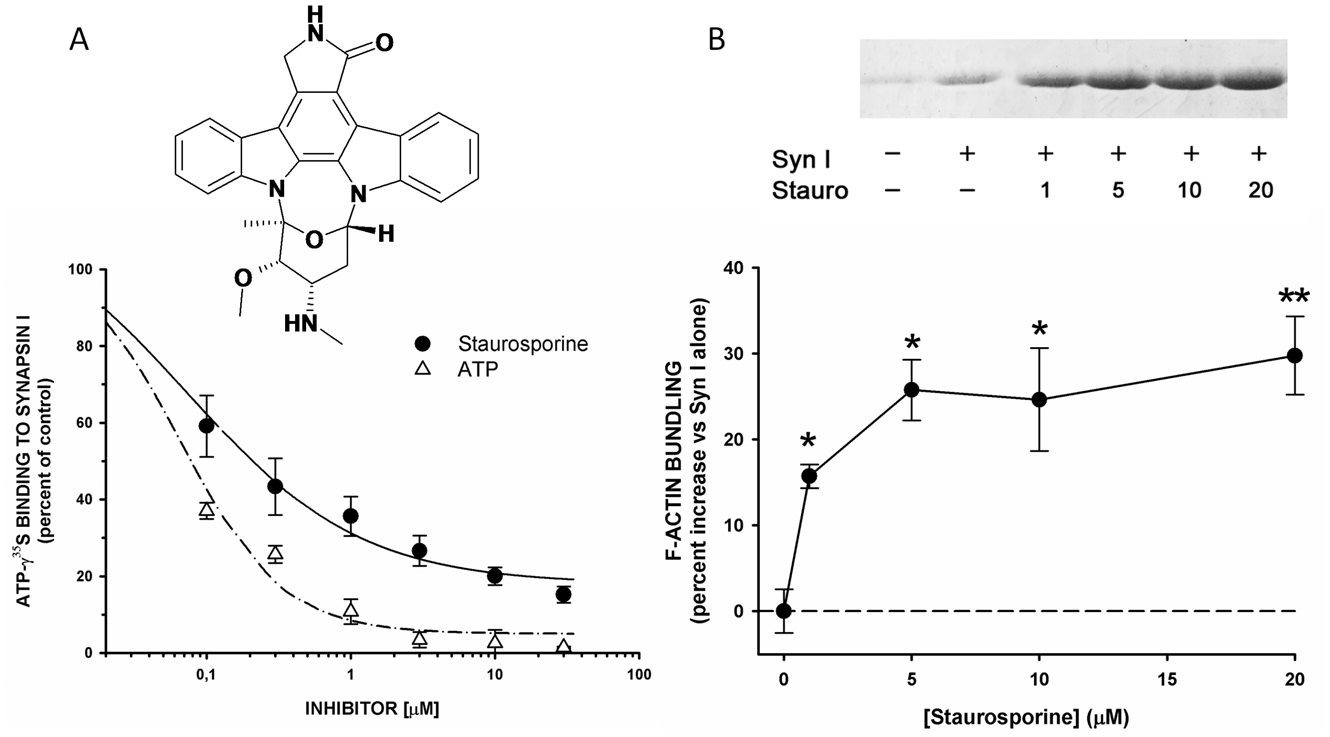
Staurosporine binds to synapsin I and affects synapsin I-dependent F-actin bundling. A. *Upper panel:* Chemical structure of staurosporine. *Lower panel :* Inhibition of ATP-γ^35^S (0.2 µM) binding to purified bovine synapsin I (0.5 µM) by increasing concentrations of either cold ATP (open triangles) or staurosporine (filled circle). The amount of ATP-γ^35^S bound in the presence of the inhibitors is expressed in percent of the binding under control conditions (absence of either inhibitor). Points in the plot are means ± sem from 5 independent experiments. Inhibition curves were fitted using a 3-parameter sygmoidal dose-response function yielding IC_50_ and lower plateau values (see Fig. 6). B. The F-actin bundling activity of synapsin I (final concentrations : synapsin I, 0.5 µM; F-actin, 5 µM) in the absence or presence of the indicated concentration of staurosporine (1–20 µM) was evaluated by using the low speed sedimentation assay. The actin recovery in the pellet was evaluated by densitometric analysis of the actin bands from Coomassie-stained gels of the solubilized pellets. A representative experiment is shown in the *upper panel* (staurosporine concentrations are shown in µM). The percent increases in actin bundling observed in the presence of increasing concentrations of staurosporine, with respect to the samples containing F-actin and synapsin I only, is shown in the *lower panel* as means ± sem from 5 independent experiments. Statistical analysis was carried out by one-way Anova followed by the post-hoc Dunnett's multiple comparison test (*, p<0.05 ; **, p<0.01).

We showed in the first series of computations (**Fig. 2A**) that ATP-binding sites of protein kinases do not resemble neither ATP-binding sites of other kinases nor other ATP-binding cavities [41], [70]. It is therefore not surprising that 123 out of the 134 binding sites (92%) scored above an acceptable similarity threshold (SiteAlign d2 score <0.2)[41] are annotated as ATP-binding sites in protein kinases (**Fig. 2A**, **Supplementary Table S1**). Out of the 11 outliers, two entries (PDB entries 1aux and 1px2) drew our attention since they both describe the ATP-binding site of synapsin I. Despite a low homology (21%) between human Pim-1 (1yhs) and bovine synapsin I (1aux) amino acid sequences, the proposed 3-D alignment between both binding sites reveal remarkable shared features. Although both proteins adopt distinct 3-D folds, their bound ligands (staurosporine in Pim-1, ATP-γS in synapsin I) in the cognate X-ray structures occupy a similar orientation in their respective binding sites (**Fig. 2B**). Out of the 32 and 24 cavity-lining residues in 1yhs and 1aux, respectively, 11 amino acids matched in both their chemical properties and 3-D spatial coordinates (**Fig. 2C**). 6 pairs of short side-chain aliphatic residues, one pair of lysine residues, two pairs of negatively-charged amino acids, one pair of glycine and one pair of serine residues are absolutely conserved in both binding sites (**Fig. 2C**). To be sure that non-conserved residues in the synapsin I site would not impair staurosporine recognition, we attempted preliminary docking experiments of the latter ligand to the 1aux structure. Only the floppy Lys67 side chain which points inward the ATP-γS binding site was rendered flexible during docking to putatively enlarge the cavity. Docking staurosporine in synapsin I with the GOLD software (see structure in **Fig. 3A**) provided a single set of similar binding poses with mostly hydrophobic intermolecular contacts and a bidentate hydrogen bond to main chain atoms of a hinge region (Pro^306^, Ile^308^; see top-ranked pose **Fig. 2D**).

### Staurosporine binds to synapsin I and enhances synapsin- F-actin interactions

The *in silico* predicted interaction between staurosporine and synapsin I was tested by *in vitro* experiments aimed at analyzing the ability of staurosporine to competitively inhibit ATPγ^35^S binding to synapsin I [53], [54] or to affect the interactions of synapsin I with actin which occur through a major binding site in domain C [71], [72], [73]. When purified bovine synapsin I was incubated with ATPγ^35^S in the absence or presence of increasing concentrations of either staurosporine or cold ATP (Fig. 3A), both ligands were able to inhibit ATPγ^35^S in a concentration-dependent fashion (IC_50_ 0.07±0.01 µM and maximal inhibition 95.8±2.2% for ATP; IC_50_ 0.31±0.09 µM and maximal inhibition 84.3±1.2% for staurosporine), indicating that staurosporine bound synapsin I at the ATP binding site.

Since the Synapsin I ATP binding pocket is localized in the synapsin domain primarily involved in actin binding [69], [72], we investigated whether staurosporine binding is able to affect the synapsin I-actin interaction. To this aim, we evaluated the F-actin binding/bundling activity under conditions of ATP binding to synapsin I in the presence of increasing concentrations of staurosporine ranging from to 1 to 20 µM. The amount of F-actin/synapsin I bundles recovered by low speed sedimentation was increased by staurosporine by approximately 30% at 5 µM (Fig. 3B), indicating that binding of the kinase inhibitor to the ATP binding site of synapsin I modifies its molecular interactions with the F-actin-based cytoskeleton.

### Synapsin I is closer to Pim-1 than to other protein kinases

Staurosporine is a pan-kinase inhibitor exhibiting not only nanomolar affinities to Pim-1 but also to a wide array of protein kinases [42]. To ascertain whether the ATP-binding site of synapsin I is equally close to all known ATP-binding sites or specifically related to Pim-1, we computed with SiteAlign [41] the distance between the ATP-binding site of bovine synapsin I (1aux) and 978 ATP-binding sites from the Protein Data Bank. The 978 ATP-binding binding sites were extracted from the sc-PDB database [39] and feature a total of 433 unique proteins among which 110 are protein kinases. 113 entries describing 46 different protein kinases present a binding site distance below 0.20, the previously-determined computed threshold for discriminating similar from dissimilar binding sites [41]. This result suggests that the ATP site of synapsin I is similar to that of many other protein kinases. When looking at the top 25 ranked entries (**Table 1**), Pim-1 binding sites to various ATP-competitive inhibitors are the most numerous (8 times), but other serine/threonine protein kinases (e.g. casein kinase II) also share strong binding site similarities with the ATP site of synapsin I. A statistical analysis of binding site distances (SiteAlign d2 distance) to that of synapsin I was undertaken by computing, for each single protein present at least in 5 copies in the sc-PDB, the area under the ROC curve [40] in a simple binary classification system (similar, dissimilar) is calculated. Briefly, each of the 978 ATP-binding sites are ranked by decreasing distance to that of synapsin I and the rank distribution of every binding site sharing the same protein name (true positives are presumed similar to the reference) are compared to the ranks of all other active sites (true negatives are presumed dissimilar to the reference, see full results in **Supplementary Table S2**).

**Table 1:** The 25 ATP-binding sites of protein kinases closest to that of bovine synapsin I (PDB entry 1aux).

PDB^a^	d2^b^	Name	HET^c^
2oxd	0.1008	Casein kinase II subunit alpha	K32
2oxx	0.1038	Casein kinase II subunit alpha	K22
1yi3	0.1056	Proto-oncogene serine/threonine-protein kinase Pim-1	LY2
1tqm	0.1083	RIO-type serine/threonine-protein kinase Rio2	ANP
3cy3	0.1176	Proto-oncogene serine/threonine-protein kinase Pim-1	JN5
3bgz	0.1199	Proto-oncogene serine/threonine-protein kinase Pim-1	VX3
3cy2	0.1233	Proto-oncogene serine/threonine-protein kinase Pim-1	MB9
2bik	0.1327	Proto-oncogene serine/threonine-protein kinase Pim-1	BI1
2c1a	0.1339	Protein kinase A	I5S
1xr1	0.1350	Proto-oncogene serine/threonine-protein kinase Pim-1	ANP
1bl6	0.1368	Mitogen-activated protein kinase 14	SB6
1q8u	0.1385	cAMP-dependent protein kinase catalytic subunit alpha	H52
3bi6	0.1388	Wee1-like protein kinase	396
3biz	0.1395	Wee1-like protein kinase	61E
2i0e	0.1400	Protein kinase C beta type	PDS
1cm8	0.1405	Mitogen-activated protein kinase 12	ANP
2uzv	0.1423	cAMP-dependent protein kinase catalytic subunit alpha	SS5
1yi4	0.1456	Proto-oncogene serine/threonine-protein kinase Pim-1	ADN
2hen	0.1467	Ephrin type-B receptor 2	ADP
2rkp	0.1474	Casein kinase II subunit alpha	RFZ
2ojg	0.1481	Mitogen-activated protein kinase 1	19A
2z7s	0.1481	Ribosomal protein S6 kinase alpha-1	P01
2csn	0.1482	Casein kinase I homolog 1	CKI
1yhs	0.1483	Proto-oncogene serine/threonine-protein kinase Pim-1	STU
1mu	0.1508	Serine/threonine-protein kinase 6	ADN

a PDB entry [10].

b SiteAlign [41] distance measuring the local similarity to the query binding site (1aux).

c Ligand Chemical Component identifier in the cognate PDB complex (http://ligand-expo.rcsb.org/ld-search.html).

**Figure 4:**
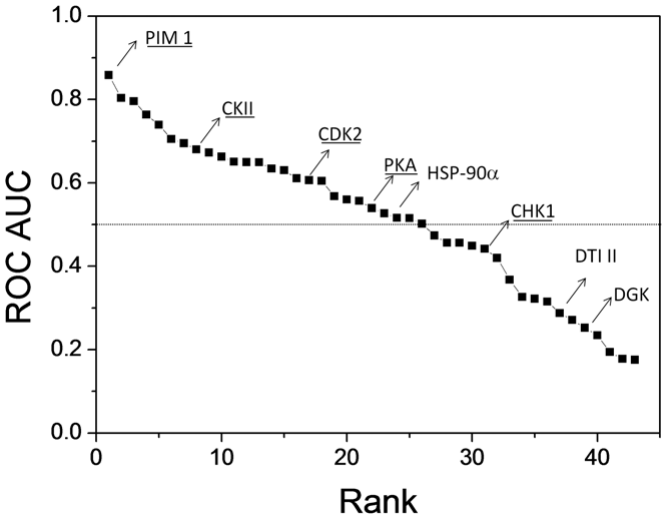
Similarity of ATP-binding proteins to bovine synapsin I. ATP-binding proteins present in at least 5 copies in the sc-PDB dataset (n = 43) are ranked by decreasing ROC score (area under the ROC curve), iteratively computed for each protein from the SiteAlign [41] alignment (d2 score) to the ATP-binding site of bovine synapsin I (PDB entry 1aux). Protein kinases are underlined. PIM 1, Proto-oncogene serine/threonine-protein kinase Pim-1; CK II, Casein kinase II subunit alpha; CDK2, Cell division protein kinase 2 (cyclin-dependent kinase 2) ; PKA, cAMP-dependent protein kinase catalytic subunit alpha (Protein kinase A); HSP-90α, Heat shock protein HSP 90-alpha; CHK1, Serine/threonine-protein kinase Chk1; DTI II, DNA topoisomerase II, DGK, Diacylglycerolkinase).

**Figure 5:**
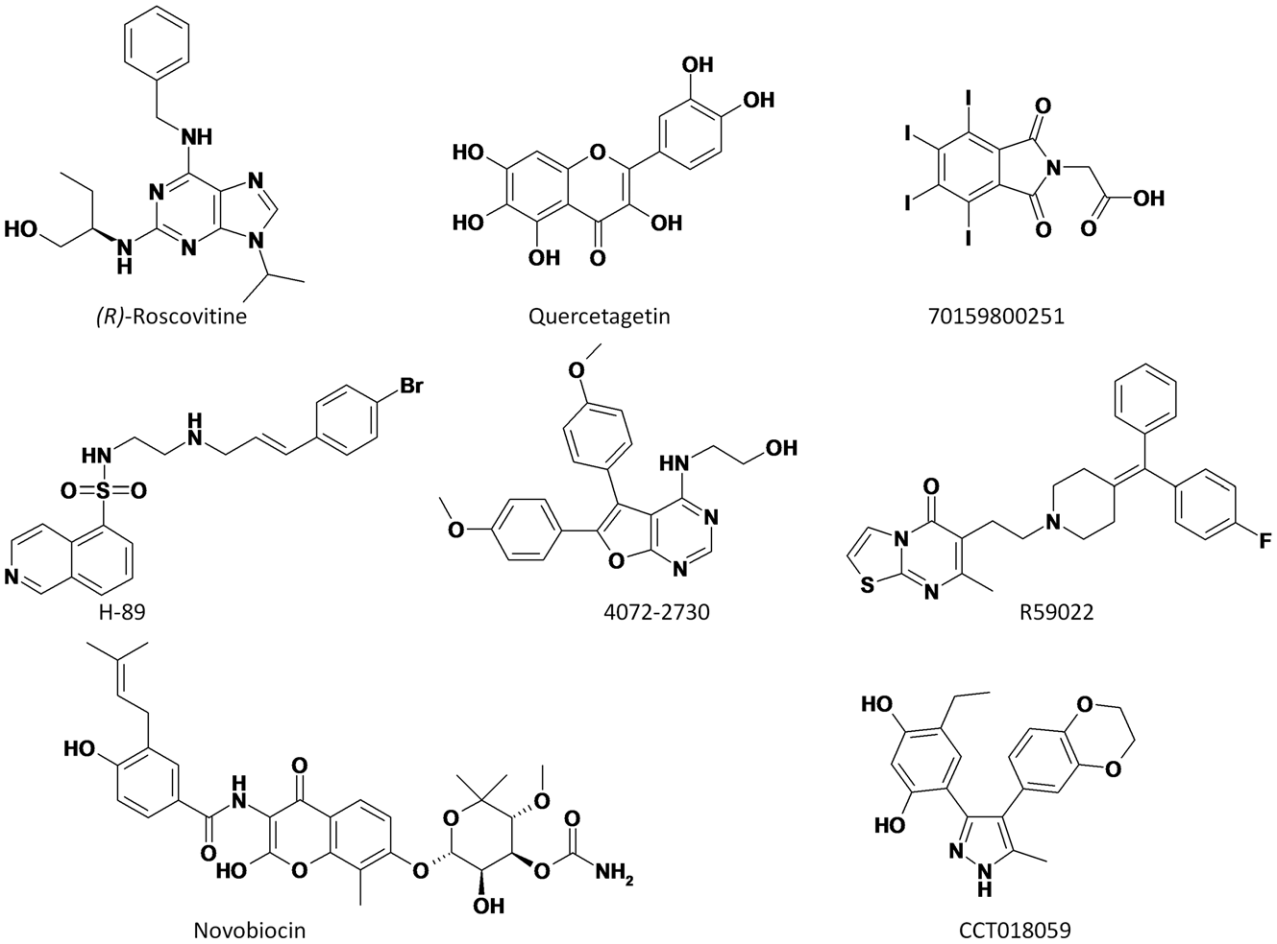
Chemical structures of compounds tested for binding to bovine synapsin I. *(R)*-Roscovitin (CDK2 inhibitor), Quercetagetin (Pim-1 kinase inhibitor), 7015980251 (Casein kinase II inhibitor), H-89 (protein kinase A inhibitor), 4072–2730 (Protein kinase Chk1 inhibitor), R59022 (diacylglycerolkinase inhibitor), Novobiocin (DNA topoisomerase II inhibitor), and CCT018159 (HSP-90 alpha inhibitor).

Areas under the curve (AUC) vary from very high (e.g. Pim-1; AUC  = 0.85) to significant (e.g. casein kinase II or CKII, cyclin-dependent kinase 2 or CDK2; AUC >0.65) to random (protein kinase A or PKA, Heat shock protein-90α or HSP-90α; AUC ≈0.50) and to even worse than random (Checkpoint kinase 1 or CHK1, DNA topoisomerase II, Diacylglycerolkinase or DGK; AUC <0.50; **Fig. 4**). We therefore predicted that inhibitors of protein active sites presenting a high ROC AUC value (Pim-1 kinase, CDK2, CKII) should cross-react with synapsin I whereas inhibitor of other proteins presenting a close-to-random of even lower AUC value (PKA, HSP-90α, CHK1, DNA topoisomerase II, DGK) should not. One high-affinity inhibitor (**Fig. 5**) of each of these 8 representative targets was purchased and tested for in vitro binding to bovine synapsin I. It is important to point out that these commercially available inhibitors and/or very close analogs have all been co-crystallized in the ATP-binding site of their respective target.

**Figure 6:**
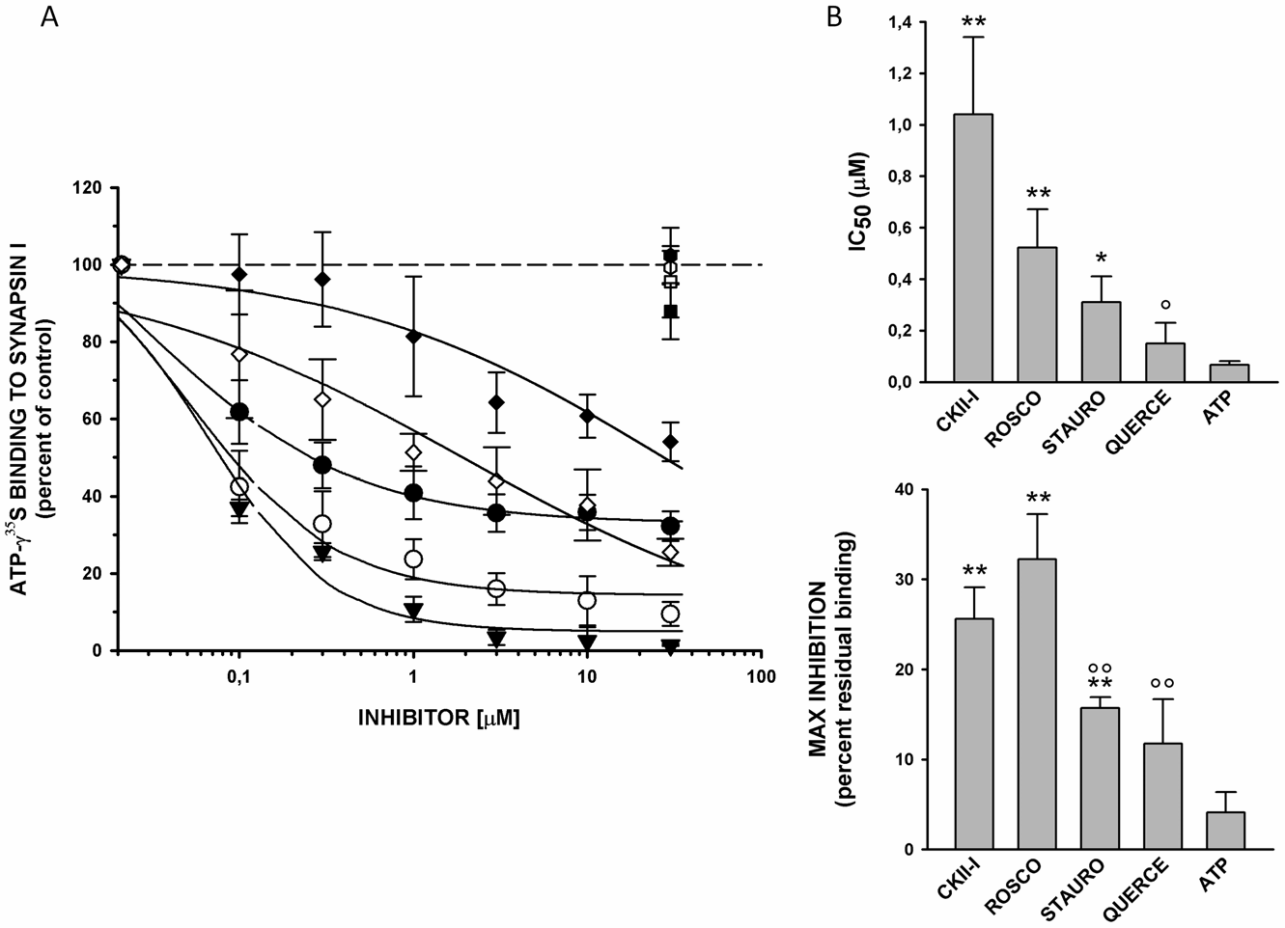
Binding of distinct ATP-competitors to the ATP-binding site of bovine synapsin I. A. Inhibition curves of ATP-γ^35^S (0.2 µM) binding to purified bovine synapsin I (0.5 µM) by increasing concentrations of either cold ATP (closed triangles), quercetagetin (open circles), roscovitin (closed circles), H-89 (closed diamond), 7015980251 (open diamond), R59022 (open hexagon), CCT018159 (closed hexagon), 4072–2730 (open square) and novobiocin (closed square). The amount of ATP-γ^35^S bound in the presence of the inhibitors is expressed in percent of the binding under control conditions (absence of either inhibitor). Points in the plot are means ± sem from 5 independent experiments. Inhibition curves were fitted using a 3-parameter sigmoidal dose-response function. B. IC_50_ (*upper panel*) and lower plateau (*lower panel*) values were calculated from individual curve fittings and are shown as means ± sem from 5 independent experiments. The values of H-89 (IC_50_ >30 µM) and the inactive compounds (R59022, CCT018159, 4072–2730 and novobiocin) are not reported. Statistical analysis was carried out by one-way Anova followed by the post-hoc Bonferroni's multiple comparison test (* p<0.05, ** p<0.01 *vs* ATP ; ° p<0.05, °° p<0.01 *vs* roscovitin). CKII-I, 70159800251; QUERCE Quercetagetin; ROSCO, (*R*)-Roscovitin; STAURO, Staurosporine.

In qualitative agreement with the predictions, the Pim-1 kinase inhibitor quercetagetin was the most potent binder to synapsin I (IC_50_0.15±0.08 µM; maximal inhibition of 88.3±4.9%; **Fig. 6A,B**). *(R)*-roscovitin and compound 70159800251 still compete with ATP for binding to bovine synapsin I but with a higher IC_50_ value (1.0 and 0.5 µM, respectively) and a lower level of maximal inhibition (ca. 70%, **Fig. 6B**). Among other inhibitors tested here, other protein kinase inhibitors (PKA and CHK1 inhibitors) did not show any significant binding to synapsin I (**Fig. 6A**).

## Discussion

Comparing at high-throughput 3-D features of protein cavities is likely to play an increasing role to guide the functional annotation of novel genomic structures as well as predicting target-based selectivity profiles for pharmacological ligands. Despite several sequence and fold-independent computational methods have been proposed to detect remote binding site similarities among unrelated proteins [37], there are very few *in silico* studies guided by binding site comparisons that successfully predicted either the function of a protein from its 3-D structure or assigned a novel macromolecular target to an existing ligand. The SOIPPA method [74] was successfully used to predict remote binding site similarities between the binding site of selective estrogen receptor modulators (SERMs) at the ERα receptor and the Sarcoplasmic Reticulum Ca2 ion channel ATPase protein (SERCA) transmembrane domain [75], thus explaining known side effects of SERMs. Likewise, the NAD binding site of the Rossmann fold and the S-adenosyl-methionine (SAM)-binding site of SAM-methyltransferases were found to be similar and consequently permitted the prediction of the cross-reactivity of catechol-*O*-methyltransferase (COMT) inhibitors (entacapone, tolcapone) with the *M.tuberculosis* enoyl-acyl carrier protein reductase (InhA) [76]. The same approach predicted two unexpected targets for a known inhibitor of *Trypasonoma brucei* RNA editing ligase [77]. Last, the PocketPicker algorithm [78] was used to detect a cavity on the surface of a APOBEC3A structure, a protein which is able to inactivate retroviral genomes. Encoding the pocket as correlation vectors enabled the comparison of a set of 1300 ligand-binding sites from the PDBBind dataset [79]. Among top scoring entries were only nucleic acid-binding pockets [80]. Point mutation of the cavity-lining residues effectively led to mutants with a reduced antiviral activity. The pocket was shown to recognize the small 5.8S RNA [80] as a preliminary step to inactivate retroviral particles.

The main reason for the paucity of predictive reports is that many 3-D site comparison tools are extremely sensitive to atomic coordinates and thus better suited to detect global than local similarities [81]. In this context, a true advantage of the SiteAlign algorithm, used in the current study to detect local similarity between ATP-binding sites of Pim-1 kinase and synapsin I, is that cavity descriptors are assigned to Cα carbon atoms thus rendering the method fuzzy enough to be relatively insensitive to variations in 3-D coordinates (e.g. rotameric state and orientation of a side chain). A good illustration of this feature is exemplified by the matching of Pim-1 to synapsin I binding sites (**Fig. 2C**) which clearly shows a good fit of several pairs of residues (Lys67 vs. Lys269, Glu121 vs. Glu305) with significantly different side chain orientations. Recently-described alignment-independent binding site comparison methods (PocketMatch [82], FuzCav [70]) focusing on protein C-α atoms also found a significant similarity score between 1yhs and 1aux active sites (PocketMatch PMscore  = 50.79; FuzCav score  = 0.160). Conversely, two state-of-the-art full atom-matching methods (SitesBase [83], SiteEngine [84]) failed in finding the same binding sites similar (SiteEngine Match score  = 18.71; no output for SitesBase). SiteEngine notably fails to fit the pair of glutamic acid residues (Glu121 vs. Glu305, recall **Fig. 2C**) diverging in the orientation of their side chains. A detailed binding site representation at the atomic level is therefore detrimental to the detection of remote similarity between synapsin I and Pim-1 kinase.

The herein predicted remote similarity between ATP-binding sites of Pim-1 kinase and synapsin I could be experimentally validated by an *in vitro* competition assay. The affinity of staurosporine, a pan-kinase inhibitor, to bovine synapsin I is about 0.3 µM (**Fig. 3**). It is thus comparable to that observed for most serine/threonine protein kinases [42]. The polypharmacological profile [85] of staurosporine may be attributed to its lipophilicity and the tendency to recognize apolar surface patches. The hypothesized binding mode to synapsin I is however remarkably reminiscent from that seen in protein kinases with a bidentate hydrogen bond to main chain atoms of a hinge region accompanying apolar interactions (**Fig. 2D**). Binding to synapsin I could be verified for other inhibitors (roscovitin, quercetagetin, 70159800251) targeting different protein kinases (cyclin-dependant kinases, Pim-1, casein kinase II), still with submicromolar affinities **(** **Fig. 5**). One might argue that a remote similarity among ATP-binding sites is trivial and that the herein presented data are not surprising. Interestingly, the ATP-binding site of synapsin I was predicted to be much more distant from that of other serine/threonine protein kinases (e.g. Chk1, PkA; **Fig. 4**) and this assumption could be verified in vitro by testing inhibitors of the latter proteins for binding to synapsin I (**Fig. 6**). Other ATP-competitors binding to a kinase (DGK) or two other targets (DNA topoisomerase II, HSP-90α) did not either bind to synapsin I (**Fig. 6A**). Our data therefore pinpoints a binding site similarity between synapsin I and some serine/threonine protein kinases but not all of them.

Off-targets for protein kinase inhibitors outside the protein kinome (e.g. glycogen phosphorylase, malate dehydrogenase, HSP-90β) have already been discovered by proteomics [86], [87], [88] although no inhibition constants have been reported. Notably, roscovitin binds to pyridoxal kinase at the pyridoxal but not the ATP-binding site [32]. We herewith supplement the list of off-targets for four protein kinase inhibitors with synapsin I. Binding affinities in an ATP-γ^35^S competition assay are remarkably high (submicromolar) and comparable to those seen for protein kinases.

Since some protein kinase inhibitors tested here actively and directly compete for the binding of ATP to synapsin I and modify the interactions of synapsin I with the actin-based cytoskeleton, it is tempting to speculate that at least some of the effects of protein kinase inhibitors on neurotransmitter release and presynaptic function are attributable to a direct binding to synapsin I and reveal new potential targets for the action of protein kinase inhibitors on synaptic transmission and plasticity.

## Materials and Methods

### Virtual screening of sc-PDB binding sites

Protein-ligand binding sites were retrieved from the 2006 release of the sc-PDB (http://bioinfo-pharma.u-strasbg.fr/scPDB), a database of 6 415 druggable protein-ligand binding sites [39] from the Protein Data Bank. A binding site is described by any amino acid for which at least one heavy atom is closer than 4.5 Å from any heavy atom of the bound pharmacological ligand. The full sc-PDB dataset was screened for similarity to the staurosporine-binding site of the Pim-1 kinase (PDB entry 1yhs) using standard settings of the SiteAlign v4.0 program. Algorithmic details of SiteAlign have been described elsewhere [41]. Briefly, eight topological and physicochemical attributes are projected from the Cα-atom of cavity-lining residues to an 80 triangle-discretized polyhedron placed at the center of the binding site, thus defining a cavity fingerprint of 640 integers. 3-D alignment is performed by moving the sphere within the target binding site while keeping the query sphere fixed. After each move, the distance of the newly described cavity descriptor is compared to that of the query, the best alignment having the minimal distance between both cavity fingerprints. Two distances are used in SiteAlign. The d1 distance is suited to measure global similarities and is a sum of normalized distances between the 8 descriptors on all indexed triangles with non-null values for either the query or the target. Previous benchmarking studies suggest that a d1 distance of 0.60 is a good threshold for discriminating similar from dissimilar binding sites [41]. The d2 distance is suited to measure local similarities and is a sum of normalized distances between the 8 descriptors on all indexed triangles with non-null values for both the query and the target. Previous benchmarking studies suggest that a d2 distance of 0.20 is a good threshold for discriminating similar from dissimilar binding sites. In the current screen, sc-PDB entries were ranked by increasing d2 distance to the 1hys query. To avoid false positives [41], the d2 distance was post-processed and set to 1.0 if the corresponding d1 distance is higher than 0.60.

Screening for binding site similarity to the ATP-binding site of bovine synapsin (PDB entry 1aux) was done as described above. The dataset of 978 ATP-binding sites was selected from the current sc-PDB database as previously described [41], [70]. SiteAlign screen was performed using standard settings and entries were ranked by increasing d2 distance with the same post-processing as previously described (d2 = 1 if d1 >0.6). A ROC score (area under the ROC curve) is computed from the distance table with an in-house Pipeline Pilot workflow [89] for each occurrence of a protein name represented by at least 5 entries in the dataset (n = 43). The higher the ROC score for a particular protein, the more similar its protein-ligand binding sites to that of the synapsin I reference. A ROC score of 0.5 indicate a random distribution of d2 scores for a particular protein.

### Automated docking of staurosporine to bovine synapsin I

3-D atomic coordinates of staurosporine were obtained by Corina v3.5[90] from a 2-D Marvin sketch [91]. Hydrogen atoms were added using standard topological rules in Sybyl v.8.0 [92] and coordinates were saved in mol2 format. Standard settings of the Gold v4.1 program [93] were used to dock staurosporine to the ATP-binding site of bovine synapsin (PDB entry 1aux) whose coordinates were retrieved from the sc-PDB databank [30]. The cavity was defined as any protein atom present in a 10 Å-radius sphere centered on the center of mass of the sc-PDB binding site. The side chain of Lys67 was considered flexible during the docking by explicit definition of 27 rotameric states from the standard Gold rotamer library. Poses were scored with the Goldscore fitness function.

### Comparison of bovine synapsin I and human Pim-1 ATP-binding sites with other binding site matching methods

ATP-binding sites of 1aux (bovine synapsin I) and 1yhs (human Pim-1 kinase) were retrieved from the sc-PDB website (http://bioinfo-pharma.u-strasbg.fr/scPDB) and compared with the in-house FuzCav algorithm with default parameters [70]. Web interfaces to SitesBase [83] (http://www.modelling.leeds.ac.uk/sb/), SiteEngine [84] (http://bioinfo3d.cs.tau.ac.il/SiteEngine/) and PocketMatch [82] (http://proline.physics.iisc.ernet.in/pocketmatch/) were used to compare the same entries. Active site detection was here achieved by specifying the chemical component HET code of the co-crystallized ligands (SAP for 1uax, STU for 1yhs).

### ATP-γ^35^S binding assays

Synapsin I was purified from bovine brain [71] and stored in liquid nitrogen in 200 mM NaCl, 25 mM TrisCl, pH 7.4. Synapsin I (500 nM) was incubated with 200 nM ATP-γ^35^S (Perkin Elmer, Waltham, MA) in 50 mM HEPES-NaOH pH 7.4, 25 mM NaCl, 0.5 mM CaCl_2_ and 2 mM MgCl_2_ for 1 h at room temperature in the absence or presence of increasing concentrations (0.1–30 µM) of either cold ATP, staurosporine (Sigma, Milan, Italy), *(R)-*roscovitin (Caiman, Ann Arbor, MI), quercetagetin (Calbiochem, San Diego, CA), 70159800251 (Otava, kiev, Ukraine), H-89 (LC Laboratories, Woburn, MA), 4072–7730 (ChemDiv, San Diego, CA), R59022 and novobiocin (MP Biochemicals, Illkirch, France) and last CCT018059 (SPI-Bio, Montigny Le Bretonneux, France). ATP-γ^35^S binding was quantified as previously described [94]. Briefly, aliquots of the samples were spotted onto squares of phosphocellulose paper (Upstate/Millipore, Billerica, MA). The paper squares were extensively washed with deionized water for 30 min, air-dried and analyzed for radioactivity by using the Perkin Elmer Cyclone Plus Phosphor Imager. After subtraction of the background values (samples with no synapsin I), data from individual competition curves were fitted with a sigmoidal dose-response function (f = min +(max–min)/(1+10∧((logEC50-x)*Hillslope)) using the Sigmaplot 8.0 software (SPSS Inc., Chicago, IL) to yield IC_50_ and maximal inhibition values. Data in the plots are the means ± sem of at least 5 independent experiments.

### Actin Bundling Assays

Actin was purified from acetone powder of rabbit skeletal muscles [95], [96] and stored in liquid nitrogen in in 2 mM Tris pH 8, 0.2 mM ATP, 0,2 mM CaCl_2_, 0.125 mM β-mercaptoethanol and 0.005% NaN_3_ (G-buffer). Before the experiments, both G-actin and synapsin I were prespun for 1 h at 4°C at 300,000× g to remove large aggregates. G-actin was polymerized at room temperature for 1 h in the presence of 100 mM KCl, 1.2 mM MgCl_2_. Synapsin I (final concentration, 0.5 µM) was preincubated with increasing concentrations (1–20 µM) of staurosporine for 1 h at room temperature in 200 mM NaCl, 25 mM TrisCl pH 7.4. Actin bundling was assessed by incubating the synapsin/staurosporine samples with F-actin (final concentration, 5 µM) under polymerization conditions (100 mM KCl, 1.2 mM MgCl_2_ in G-buffer) for 1.5 h at room temperature followed by low-speed centrifugation (10,000× *g* for 15 min) to separate actin bundles (Bahler & Greengard, 1987). Pellets were solubilized in sample buffer [97] and analyzed by sodium dodecylsulfate polyacrylamide gel electrophoresis (SDS-PAGE) using 10% acrylamide in the resolving gel. Gels were fixed, stained with Coomassie Blue and destained. Densitometric analysis of the actin bands was carried out by using the ImageQuant system (GE Healthcare) followed by densitometric analysis of the fluorograms and by data interpolation into a standard curve of purified G-actin run in parallel with the unknown samples.

## Supporting Information

Table S1 (0.04 MB DOC)

Table S2 (0.06 MB DOC)
