# Correction: Binding of Protein Kinase Inhibitors to Synapsin I Inferred from Pair-Wise Binding Site Similarity Measurements

**DOI:** 10.1371/annotation/95078306-dc3b-4441-b2b1-dd986b734570

**Published:** 2010-10-12

**Authors:** Enrico Defranchi, Claire Schalon, Mirko Messa, Franco Onofri, Fabio Benfenati, Didier Rognan

The first author's name is misspelled. The correct spelling is: Enrico Defranchi. The correct citation is: Defranchi E, Schalon C, Messa M, Onofri F, Benfenati F, et al. (2010) Binding of Protein Kinase Inhibitors to Synapsin I Inferred from Pair-Wise Binding Site Similarity Measurements. PLoS ONE 5(8):e12214.doi:10.1371/journal.pone.0012214. The author contributions should read: Conceived and designed the experiments: FB DR. Performed the experiments: ED CS MM FO. Analyzed the data: ED CS MM FO DR. Wrote the paper: FB DR.

