# Supplementary material for: Binding of Protein Kinase Inhibitors to Synapsin I Inferred from Pair-Wise Binding Site Similarity Measurements
Source: PLoS One. 2010 Aug 16;5(8):e12214. doi: 10.1371/journal.pone.0012214 (PMC2922380; doi:10.1371/journal.pone.0012214)
Supplement: Table S1 — (0.04 MB DOC) [file pone.0012214.s001.doc]

**Table S1.** sc-PDB protein-ligand binding sites ranked by decreasing SiteAlign [39] distance to the ATP-binding site of human Pim-1 kinase (1yhs ). Only entries distant by less than 0.2 are listed. Proteins devoid of kinase activity are listed in bold.

**PDB^a^ d2^b^ Name**

1yxx 0.019676 Proto-oncogene serine/threonine-protein kinase Pim-1

1yxv 0.026157 Proto-oncogene serine/threonine-protein kinase Pim-1

1yi3 0.047222 Proto-oncogene serine/threonine-protein kinase Pim-1

1p4f 0.055263 Death-associated protein kinase 1

1xr1 0.057937 Proto-oncogene serine/threonine-protein kinase Pim-1

1q41 0.063426 Glycogen synthase kinase-3 beta

1yxu 0.073016 Proto-oncogene serine/threonine-protein kinase Pim-1

1ig1 0.073438 Death-associated protein kinase 1

1h0w 0.078472 Cell division protein kinase 2

1uu9 0.081458 3-phosphoinositide-dependent protein kinase 1

1q3d 0.090476 Glycogen synthase kinase-3 beta

1h1w 0.090476 3-phosphoinositide-dependent protein kinase 1

1ydr 0.092083 cAMP-dependent protein kinase catalytic subunit alpha

1oky 0.092333 3-phosphoinositide-dependent protein kinase 1

1p2a 0.092593 Cell division protein kinase 2

1l3r 0.094445 cAMP-dependent protein kinase catalytic subunit alpha

1yds 0.098125 cAMP-dependent protein kinase catalytic subunit alpha

2bik 0.100198 Proto-oncogene serine/threonine-protein kinase Pim-1

1q8w 0.101535 cAMP-dependent protein kinase catalytic subunit alpha

1uu8 0.101587 3-phosphoinositide-dependent protein kinase 1

1r0e 0.104348 Glycogen synthase kinase-3 beta

1q97 0.104902 Serine/threonine-protein kinase SKY1

1szm 0.107197 cAMP-dependent protein kinase catalytic subunit alpha

2brm 0.107765 Serine/threonine-protein kinase Chk1

1j1c 0.110417 Glycogen synthase kinase-3 beta

2biy 0.113542 3-phosphoinositide-dependent protein kinase 1

2brg 0.115972 Serine/threonine-protein kinase Chk1

1yi4 0.116468 Proto-oncogene serine/threonine-protein kinase Pim-1

1ydt 0.116840 cAMP-dependent protein kinase catalytic subunit alpha

1yw2 0.118333 Mitogen-activated protein kinase 14

2src 0.119965 Proto-oncogene tyrosine-protein kinase Src

1jkl 0.120290 Death-associated protein kinase 1

1oiq 0.120833 Cell division protein kinase 2

1u59 0.122917 Tyrosine-protein kinase ZAP-70

1xbc 0.123370 Tyrosine-protein kinase SYK

1oit 0.124008 Cell division protein kinase 2

1pkd 0.124432 Cell division protein kinase 2

1pjk 0.124479 Casein kinase II

2brb 0.124621 Serine/threonine-protein kinase Chk1

1mq4 0.124840 Serine/threonine-protein kinase 6

1ql6 0.125521 Phosphorylase kinase

1xbb 0.125625 Tyrosine-protein kinase SYK

1o6k 0.126833 Glycogen synthase kinase-3 beta

2bhe 0.127315 Cell division protein kinase 2

1j1b 0.128846 Glycogen synthase kinase-3 beta

1y6a 0.129861 Vascular endothelial growth factor receptor 2

1pxj 0.129960 Cell division protein kinase 2

1tqm 0.130000 RIO-type serine/threonine-protein kinase Rio2

1v1k 0.131349 Cell division protein kinase 2

1pxl 0.132018 Cell division protein kinase 2

2bfy 0.132118 Serine/threonine-protein kinase 12-A

1atp 0.132692 cAMP-dependent protein kinase catalytic subunit alpha

1e1v 0.132708 Cell division protein kinase 2

1h1q 0.132937 Cell division protein kinase 2

1rej 0.134722 cAMP-dependent protein kinase catalytic subunit alpha

1rek 0.135494 cAMP-dependent protein kinase catalytic subunit alpha

1ir3 0.136775 Insulin receptor

1vyz 0.136979 Cell division protein kinase 2

1gii 0.137083 Cell division protein kinase 2

1yqj 0.137500 Mitogen-activated protein kinase 14

1svg 0.139410 cAMP-dependent protein kinase catalytic subunit alpha

1pxi 0.140079 Cell division protein kinase 2

1pyx 0.141346 Glycogen synthase kinase-3 beta

1uu3 0.141477 3-phosphoinositide-dependent protein kinase 1

1qpc 0.141477 Proto-oncogene tyrosine-protein kinase LCK

1jsv 0.142647 Cell division protein kinase 2

1mp8 0.143254 Focal adhesion kinase 1

1phk 0.144565 Phosphorylase b kinase gamma catalytic chain, skeletal muscle isoform

1h1r 0.145040 Cell division protein kinase 2

1ymi 0.145040 Casein kinase II

1ouy 0.145644 Mitogen-activated protein kinase 14

1qpd 0.146032 Proto-oncogene tyrosine-protein kinase LCK

1e1x 0.147588 Cell division protein kinase 2

1q99 0.147727 Serine/threonine-protein kinase SKY1

1q8t 0.148295 cAMP-dependent protein kinase

1y8y 0.149781 Cell division protein kinase 2

1h0v 0.150000 Cell division protein kinase 2

1unl 0.151705 Cell division protein kinase 5

2bro 0.152174 Serine/threonine-protein kinase Chk1

2brh 0.152579 Serine/threonine-protein kinase Chk1

1tqp 0.152778 RIO-type serine/threonine-protein kinase Rio2

1oec 0.153125 Fibroblast growth factor receptor 2

1ol5 0.153333 Aurora-A

1b38 0.154545 Cell division protein kinase 2

2br1 0.154792 Serine/threonine-protein kinase Chk1

1v0p 0.155357 Cell division control protein 2 homolog

1fin 0.156061 Cell division protein kinase 2

1dm2 0.157738 Cell division protein kinase 2

1xjd 0.159295 Protein kinase C theta type

1u5r 0.159896 Serine/threonine-protein kinase TAO2

1jkk 0.160833 Death-associated protein kinase 1

1ke7 0.161413 Cell division protein kinase 2

1e9h 0.163068 Cell division protein kinase 2

1bmk 0.163158 Mitogen-activated protein kinase 14

1o6y 0.163622 Serine/threonine-protein kinase pknB

1fmo 0.164674 cAMP-dependent protein kinase catalytic subunit alpha

1pye 0.164683 Cell division protein kinase 2

1day 0.165667 Casein kinase II subunit alpha

1a5b 0.165972 **TRp synthase**

1u8y 0.166042 **Ras-related protein Ral-A**

1csn 0.166667 Casein kinase I homolog 1

1o6l 0.166833 RAC-beta serine/threonine-protein kinase

1u7e 0.168750 cAMP-dependent protein kinase catalytic subunit alpha

1qmz 0.168750 Cell division protein kinase 2

1aux 0.169676 **Synapsin I**

1di8 0.171825 Cell division protein kinase 2

1qpj 0.172283 Proto-oncogene tyrosine-protein kinase LCK

1gz8 0.173542 Cell division protein kinase 2

1ke6 0.173843 Cell division protein kinase 2

1cm8 0.174603 Mitogen-activated protein kinase 12

1mqb 0.175794 Ephrin type-A receptor 2

1stc 0.175868 cAMP-dependent protein kinase catalytic subunit alpha

1vrl 0.176225 **MutY adenine glycosylase**

1jqh 0.176630 Insulin-like growth factor 1 receptor kinase

1px2 0.179386 **Synapsin I**

1xws 0.179924 Proto-oncogene serine/threonine-protein kinase Pim-1

1gij 0.183750 Cell division protein kinase 2

1daw 0.184058 Casein kinase II

1n6o 0.186508 **Ras-related protein Rab-5A**

1h1s 0.186667 Cell division protein kinase 2

1b39 0.187833 Cell division protein kinase 2

1pmn 0.189375 Mitogen-activated protein kinase 10

1hck 0.189410 Cell division protein kinase 2

1pmq 0.190167 Mitogen-activated protein kinase 10

1sn0 0.191228 **Transthyretin**

1k3a 0.191486 Insulin-like growth factor 1 receptor

1i44 0.193056 Insulin receptor tyrosine kinase

1l4l 0.194444 **Nicotinate-nucleotide--dimethylbenzimidazole phosphoribosyltransferase**

1ke9 0.194907 Cell division protein kinase 2

1ktc 0.195175 **alpha-N-acetylgalactosaminidase**

1j7l 0.195625 **Aminoglycoside 3'-phosphotransferase**

1km1 0.198016 **Orotidine 5'-phosphate decarboxylase**

^a^ PDB entry

^b^ SIteAlign d2 distance
