# Supplementary material for: Binding of Protein Kinase Inhibitors to Synapsin I Inferred from Pair-Wise Binding Site Similarity Measurements
Source: PLoS One. 2010 Aug 16;5(8):e12214. doi: 10.1371/journal.pone.0012214 (PMC2922380; doi:10.1371/journal.pone.0012214)
Supplement: Table S2 — (0.06 MB DOC) [file pone.0012214.s002.doc]

**Table S2.** ATP-binding proteins ranked by decreasing ROC score (area under the ROC curve) computed from SiteAlign d2 distances from the bovine synapsin I (PDB entry 1aux) ATP binding site. Only proteins present in at least 5 copies have been taken into account here.

| **Protein** | **ROC score** | **Number of entries** |
| --- | --- | --- |
| Proto-oncogene serine/threonine-protein kinase Pim-1 | 0.86 | 14 |
| Serine/threonine-protein kinase 6 | 0.80 | 15 |
| Dual specificity mitogen-activated protein kinase kinase 1 | 0.80 | 5 |
| Serine/threonine-protein kinase pknB | 0.76 | 5 |
| Biotin carboxylase | 0.74 | 12 |
| Wee1-like protein kinase | 0.70 | 6 |
| Mitogen-activated protein kinase 1 | 0.69 | 9 |
| Casein kinase II subunit alpha | 0.68 | 22 |
| Protein tyrosine kinase 2 beta | 0.67 | 5 |
| 3-phosphoinositide-dependent protein kinase 1 | 0.66 | 11 |
| Insulin receptor | 0.65 | 5 |
| Glycogen synthase kinase-3 beta | 0.65 | 12 |
| Phosphoglycerate kinase 1 | 0.65 | 7 |
| Phosphatidylinositol-4,5-bisphosphate 3-kinase catalytic subunit γ | 0.63 | 13 |
| Endoplasmin | 0.63 | 10 |
| Focal adhesion kinase 1 | 0.61 | 10 |
| Cyclin-dependant kinase 2 | 0.61 | 103 |
| Vascular endothelial growth factor receptor 2 | 0.60 | 14 |
| Hepatocyte growth factor receptor | 0.57 | 5 |
| Mitogen-activated protein kinase 14 | 0.56 | 42 |
| Mitogen-activated protein kinase 10 | 0.56 | 11 |
| Protein kinase A | 0.54 | 49 |
| Proto-oncogene tyrosine-protein kinase ABL1 | 0.53 | 14 |
| Heat shock protein HSP 90-alpha | 0.52 | 35 |
| Probable inorganic polyphosphate/ATP-NAD kinase 1 | 0.52 | 6 |
| ATP-dependent molecular chaperone HSP82 | 0.50 | 12 |
| Nucleoside diphosphate kinase | 0.47 | 17 |
| Pyridoxal kinase | 0.46 | 6 |
| Actin-related protein 3 | 0.46 | 5 |
| Proto-oncogene tyrosine-protein kinase LCK | 0.45 | 10 |
| Serine/threonine-protein kinase Chk1 | 0.44 | 24 |
| NTPase P4 | 0.42 | 5 |
| DNA ligase | 0.37 | 6 |
| ATP synthase subunit alpha | 0.33 | 6 |
| Proto-oncogene tyrosine-protein kinase Src | 0.32 | 17 |
| Poly(A) polymerase, putative | 0.31 | 5 |
| DNA topoisomerase II | 0.29 | 12 |
| Ribonuclease pancreatic | 0.27 | 27 |
| Diacylglycerolkinase | 0.25 | 13 |
| Heat shock cognate 71 kDa protein | 0.23 | 10 |
| Phosphoenolpyruvate carboxykinase | 0.19 | 8 |
| DNA polymerase iota | 0.18 | 5 |
| Pantothenate synthetase | 0.18 | 7 |
